# Spray-Flame Synthesis of NASICON-Type Rhombohedral (α) Li_1+x_Y_x_Zr_2−x_(PO_4_)_3_ [x = 0–0.2] Solid Electrolytes

**DOI:** 10.3390/nano14151278

**Published:** 2024-07-30

**Authors:** Md Yusuf Ali, Tianyu Chen, Hans Orthner, Hartmut Wiggers

**Affiliations:** 1 Institute for Energy and Materials Processes—Reactive Fluids, University of Duisburg-Essen, 47057 Duisburg, Germany; yusuf.ali@uni-due.de (M.Y.A.); tianyu-chen@marubeni.com (T.C.); hans.orthner@uni-due.de (H.O.); 2 CENIDE, Center for Nanointegration Duisburg-Essen, 47057 Duisburg, Germany

**Keywords:** spray-flame synthesis, solid-state electrolyte, NASICON type Li_1+x_Y_x_Zr_2−x_(PO_4_)_3_, nanoparticles, Li-ion batteries

## Abstract

Since solid electrolytes have a broad electrochemical stability window, are exceptionally electrochemically stable against Li metal, and function as a physical separator to prevent dendrite growth, they are at the forefront of alternate possibilities, further increasing the stability and energy density of Li-ion batteries. NASICON-type electrolytes are a promising candidate due to their negligible moisture sensitivity, which results in outstanding stability and a lower probability of Li_2_CO_3_ passivity under the ambient atmosphere. However, one of the most promising representatives, Li_1+x_Y_x_Zr_2−x_(PO_4_)_3_ (LYZP), has multiple stable phases with significant variation in their corresponding Li-ion conductivity. In this paper, we have successfully synthesized the highly ionically conductive rhombohedral phase of LYZP via spray-flame synthesis. Two different solvent mixtures (e.g., 2-ethyl hexanoic acid/ethanol, propanol/propanoic acid) were chosen to explore the effect of precursor composition and combustion enthalpy on the phase composition of the nanoparticle. The as-synthesized nanoparticles from spray-flame synthesis consisted of the crystalline tetragonal zirconia (t-ZrO_2_) phase, while lithium, yttrium, and phosphate were present on the nanoparticles’ surface as amorphous phases. However, a short annealing step (1 h) was sufficient to obtain the NASICON phase. Moreover, we have shown the gradual phase conversion from orthorhombic β phase to rhombohedral α phase as the annealing temperature increased from 700 °C to 1300 °C (complete removal of β phase). In this context, Y^3+^ doping was also crucial, along with the appropriate solvent mixture and annealing temperature, for obtaining the much-desired rhombohedral α phase. Further, 0.2 at% Y^3^+ doping was added to the solvent mixture of 2-ethyl hexanoic acid/ethanol, and annealing at 1300 °C for 1 h resulted in a high ionic conductivity of 1.14∙10^−5^ S cm^−1^.

## 1. Introduction

Electrical energy has become an indispensable part of our modern civilization. However, the significant origin of the same is still from nonrenewable sources. Moreover, the energy market will remain volatile in the future due to constant depletion and geopolitical instabilities [1,2]. Consequently, sustainable energy resources such as wind, solar, bioenergy, and hydropower come to the center of the stage [3,4,5]. Nevertheless, no matter how “low carbon” our market is, or furthermore, no matter how advanced and considerate our energy management system is [6], the fluctuation of energy usage plays a part in Achilles’ heel. Amongst systems in the field of battery energy storage [7], solutions for serving energy support based on rechargeable batteries, mainly lithium-ion batteries (LIBs), have become the spotlight of this stage [7]. In this context, energy density, storage capacity, and thermal sustainability play an important role in establishing the next-best energy storage system. Since the term LIB has been available in the market since the late 1960s [8] and successfully commercialized in the 1990s [8,9], LIB storage systems have gained increasing interest in numerous application scenarios. However, employing high-energy-density Li metal anodes with conventional liquid or polymer electrolytes is not considered a ‘termination’ of lithium anodes for high-energy batteries. Regarding strict requirements of heat management [10,11,12], solid electrolytes (SEs) (e.g., inorganic [13,14,15], polymer [16], and hybrid composites [17], etc.) are believed to overcome obstacles due to their robust mechanical strength and high Li-ion transference number [18,19,20,21]. In addition, compared with liquid organic electrolytes, SEs have a wide electrochemical potential window of up to 5 V for battery cycling [22], which could empower the electric vehicle revolution and stationary demands. Employing solid composite electrolytes (SCE) could eliminate polymer-based mechanical separators, avoiding the risk of flammability and Li dendrite growth [23]. Moreover, the good wetting ability of solid electrolytes by the metallic lithium anode can reduce or even avoid the lithium dendrite formation in all-solid-state Li-metal batteries, resulting in high energy-density batteries [24]. Amongst many inorganic solid electrolytes, perovskite-type Li_3x_La_2/3−x_TiO_3_ (LLTO) [25], garnet-type Li_7_La_3_Zr_2_O_12_ (LLZO) [26], NASICON-type Li_1+x_Al_x_Ti_2−x_(PO_4_)_3_ (LATP) [27], and anti-perovskite structure Li_2_OHX (X = Cl, Br) [28] exhibit high Li-ion conductivity up to >10^−4^ S·cm^−1^ at room temperature. However, there are also challenges when using inorganic solid electrolytes. Y. Shimonishi et al. and Y. Li et al. [29,30] demonstrated the instability of garnet-type SEs in moist air (e.g., Li_7−x_La_3_Zr_2_O_12−(1/2x)_). So is the anti-perovskite structure of Li_3_OB formation [31]. The chemical reaction between those SEs and moisture causes structural damage to solid electrolytes [32]. Consequently, the Li-ion conductivity of ceramics decreases, resulting in a significant increase in Li-ion interface impedance. On the other hand, NASICON-type and perovskite-type oxides containing Ti^4+^ have shown high stability in water [33].

NASICON is an acronym for sodium (Na) super-ionic conductor with the chemical formula A_1_B_2_(PO_4_)_3_, where the A site is occupied by a monovalent cation and the B site is either a single tetravalent, or a combination of tri, tetra, and pentavalent ions [34]. One potential Li-ion conducting candidate of the NASICON family is LiZr_2_(PO_4_)_3_ (LZO), which is electrochemically stable in contact with lithium metal [35]. The ionic conduction of LZO is lower than that of its analogs, LiGe_2_(PO_4_)_3_ (LGP) and LiTi_2_(PO_4_) (LTP). It is a known fact that due to Ti^4+^ reduction in the presence of a Li anode, the use of LTP is very limited [36,37]. On the other hand, the cost of Ge precursors is generally relatively high, which makes affordable SEs obsolete [38]. However, Zr^4+^ demonstrates good chemical stability against a Li anode [39], as the energy of the Zr^4+^/Zr^0^ in LiZr_2_(PO_4_)_3_ is above the Fermi level of Li metal [40]. An obstacle to NASICON-type LZO is its polymorphism with four crystalline forms (α and α′, β and β′ phase) [41,42,43]. Replacing tetravalent Zr^4+^ with trivalent metal ions (e.g., Y^3+^) can influence the stability of lithium metal and increase the ionic conductivity above 10^−5^ S·cm^−1^ in accounting for mobile ions [44]. It has been shown that, depending upon the synthesis route and reaction temperature, NASICON-type LZO undergoes considerable phase transformations. According to Li et al. [44], the rhombohedral α phase (space group R-3c) transfers to the triclinic α’ phase at temperatures below 60 °C. On the other hand, the orthorhombic β phase transfers to the monolithic β’ phase at a higher temperature (~300 °C). The goal of this work is to synthesize the rhombohedral α phase, which is stable at room temperature with the highest ionic conductivity (above 10^−5^ S cm^−1^) compared to its polymorph phases (e.g., triclinic α’), and to avoid the presence of the β phase.

Synthesis methods involving multiple pre- and post-processing steps are primary in determining SE characteristics. Nano-size materials have been shown to have size-induced high ionic conductivity and a high surface area with excellent mechanical properties [26,45]. The rising interest in nanosolid electrolytes is prompting the search for better synthesis technology. The commonly used high-temperature solid-state reaction of suitable starting materials requires an excellent mixture to form a homogeneous precipitation and, consequently, a time-consuming calcination and/or sintering process at over 1000 °C [46,47]. Nevertheless, it might result in undesired powder due to using course materials in the micrometer-size range [48,49]. In contrast, the sol-gel method based on dissolved precursors with subsequent annealing doesn’t require that high energy; however, impurities in other phases seem inevitable [50]. Accordingly, the performance of the final product [51] is strongly influenced by the starting material [52,53]. Therefore, a highly versatile and flexible production process with high throughput is required that is also capable of providing materials with high purity. Hence, the choice of a suitable synthesis route is, therefore, decisive for reliable nanoparticle production that enables the manufacture of high-quality materials.

As an inexpensive manufacturer of ceramics, especially ceramics at the nanoscale, flame synthesis technology has shown its distinguishing characteristics [54]. Considering the homogeneity of particles and the predetermination of the composition of multinary metal oxides, we focus on gas-phase-based spray-flame synthesis (SFS). By applying a sprayer or an atomizer [55], the limited utilization of volatile and cost-effective vaporizable precursors such as organic metal salts is unlocked. Since the successful establishment of SFS in 2002 [56], this route has gained increasing interest. SFS has proven its versatile module application sharing routes with plasma, laser, and electrically heated wall reactors [57,58,59]. The merits of applying for SFS are self-explanatory. In some cases, the properties of materials in combination with a suitable thermal post-treatment, such as control over particle size (e.g., nm to µm), crystallinity, morphology and shape, and phase composition of products, can be well controlled [60]. Unlike batch processes, i.e., time-consuming sol-gel synthesis requiring pre- and post-processing steps such as gelation and drying, SFS based on spraying a solution containing dissolved precursors allows fast and continuous production.

In this work, our focus was to synthesize rhombohedral α phase NASICON Li_1+x_Y_x_Zr_2−x_(PO_4_)_3_ (LYZP) with the highest phase purity possible. Due to the benefits stated above, SFS was chosen as the synthesis route. As this is the very first (to the best of our knowledge) report to synthesize NASICON-type LYZP nanoparticles via SFS, based on literature [61,62], we investigated their synthesis using two different solvent mixtures that have been used in the past for spray-flame synthesis of metal oxides, i.e., propanol/propionic acid (PrOH/PA) and ethanol/2-ethyl hexanoic acid (EtOH/2-EHA). Furthermore, we also optimized Y^3+^ doping in LYZP to observe the effect of doping on phase purity and subsequent ionic conductivity.

## 2. Materials and Methods

In our prior experience [63], the Zr-based system does not directly produce a multicomponent final product by SFS. This is probably due to the first nucleation of ZrO_2_ in the vapor phase rather than the rest of the elements. As a result, the remaining elements (Y, Li, P) are generally present in a synthesized sample as an amorphous phase on ZrO_2_ nanoparticles. Furthermore, due to the similar phase transformation (cubic to monoclinic) of ZrO_2_ at elevated temperatures, the successful stabilization of the cubic phase of ZrO_2_ at room temperature was accomplished by Y^3+^ doping [64,65]. It has been widely studied that Y^3+^ ions replace Zr^4+^ at the cationic sublattice [65], resulting in oxygen vacancies. This directly affects the oxygen ion conductivity of ZrO_2_ and makes it appropriate for applications such as solid oxide fuel cells and sensors [66]. The stated observations are one of the major factors in synthesizing Y^3+^ undoped (LZP) and doped LZP (Li_1+x_Y_x_Zr_2−x_(PO_4_)_3_ with x = 0.1 (LY_0.1_ZP) and 0.2 (LY_0.2_ZP), respectively), to not only effect the desired phase compositions but also alleviate the ionic conductivity. Like the conventional synthesis routes [41,44,67,68], SFS also has a substantial and reasonable combination of operating parameters. Regarding the following criteria: (i) solubility of precursors in solution; (ii) melting/boiling points of precursors and solvents; and (iii) costs of precursors and solvents, we decided on suitable precursors and solvents. LiNO_3_ and Y(NO_3_)_3_ were applied as the sources of lithium and yttrium since they are both miscible in organic solvents and inexpensive. According to Lieber et al. [69], a higher metal precursor melting/decomposition temperature point relative to the solvent boiling point, i.e., T_bp_ (solvent)/T_d/mp_(precursor) less than one generates inhomogeneous particles. Moreover, their ratio values between the boiling point/decomposition of solvents (e.g., T_bp_ of 2-EHA is 217 °C) and melting point of precursors (e.g., T_mp_ of Li(NO_3_)_3_ is 264 °C, T_mp_ of Y(NO_3_)_3_ is 52 °C, Table S1) satisfy the criterion to some extent for the synthesis of homogenous particles at the nanoscale [70].

Tributyl phosphate is favored due to its low cost and solubility in our preferred solvents. As for the Zirconium precursors, a list of suitable candidates concludes zirconium nitrate (Zr(NO_3_)_4_, abbreviated as “ZN”; decomposes at 100 °C), zirconium acetate (Zr^x+^·xH_3_CCOOH, abbreviated as “ZA”, 16 wt.% diluted in acetic acid), and zirconium(IV) propoxide (Zr(OCH_2_CH_2_CH_3_)_4_, abbreviated as “ZP”, 70 wt.% in 1-propanol; T_bp_ = 208 °C). In this study, we chose ZP as a Zr precursor because, unlike ZN, can cause explosions [71] while ZP is relatively safer to use. Although metal acetate precursors are verified to result in small nanoparticles [72], ZA, only obtainable in dissolved and thus diluted form (15.0–17.0 wt% in acetic acid), has some drawbacks regarding the energy supply for combustion (low enthalpy of combustion of acetic acid with 15.32 kJ/mL). Considering all the above arguments, ZP has the highest potential regarding the above-mentioned criteria, while ZN and ZA were discarded.

As briefly stated above, depending upon the melting point/boiling point of the solvent and solute, the solvent mixture is expected to affect the synthesized particles (e.g., size, shape, surface characteristics, etc.). In this study, the chosen precursor solutions are denoted as (LY_x_ZP)_PA50_ (x = 0.2) involving solvent mixture ‘A’, i.e., propanol and propionic acid (1:1 by volume), and (LY_x_ZP)_EA50_ (x = 0.2) involving solvent mixture ‘B’, i.e., ethanol and 2-ethylhexanoic acid (1:1 by volume), as shown in Table 1. To study the maximum effect of Y doping in SFS and subsequent calcination, x = 0.2 (i.e., Y content at 20%) was chosen.

Precursors were used as supplied: LiNO_3_ (VWR, ≥99.0% purity; Leuven, Belgium), Y(NO_3_)_3_·6H_2_O (Aldrich, ≥99.8% purity; Darmstadt, Germany), Zirconium-tetra-propoxide (ZP, 70 wt% in 1-propanol, Sigma Aldrich; Darmstadt, Germany), and tributyl phosphate ((C_4_H_9_)_3_PO, TBP, Sigma Aldrich, ≥99% purity; Darmstadt, Germany). Solvent mixtures (A) and (B) were prepared from 2-propanol (PrOH, BASF, ≥99.5% purity; Ludwigshafen, Germany) and propionic acid (PA, Acros Organics, ≥99% purity; Geel, Belgium), ethanol (EtOH, VWR, ≥99.9% purity; Leuven, Belgium), and 2-ethylhexanoic acid (2-EHA, Alfa Aesar, ≥99% purity; Haverhill, MA, USA), respectively. Moreover, to compensate for the loss of lithium during synthesis and subsequent annealing [73], lithium content was increased by 50 wt.% in all experiments. The total precursor concentration was 0.5 M in all cases.

The reactor used in this research work has been described in our previous work [63]. Details of gas flow parameters and reaction pressure are listed in Table 2. The yield of as-synthesized particles is around 1 g for each experiment. For further annealing, as-synthesized particles were pressed into pellets (diameter: 5 mm, pressure: 15 kN for 15 min). A horizontal tube furnace (Carbolite Tube Furnace, type: MTF 12/38/400) was used for annealing the pellets at 700 °C and 1000 °C for 1 h under oxygen flow (6 slm) [74]. Furthermore, for annealing at higher temperatures (1150 °C and 1300 °C), a TGA (NETZSCH STA 449 F1 Jupiter) (heating rate: 10 K/min, dwelling time: 1 h, gas flow: O_2_ [44]) was used.

Powder diffraction XRD patterns were measured using an X-ray diffractometer (Malvern Empyrean diffractometer PANalytical with Cu Kα radiation). Transmission electron microscopy (TEM, Jeol JEM-2200FS; Japan) was used for particle morphology, size, and structure determination. The surface areas of the as-synthesized powders were measured using a Brunauer–Emmett–Teller (BET) device (Nova 2200, 3P Instruments GmbH; Odelzhausen, Germany). XPS spectra were recorded using a VersaProbe II (ULVAC-PHI, Chanhassen, MN, USA) equipped with Al Kα radiation. Simultaneous thermal analysis (STA) consisting of thermogravimetric analyses (TGA) and differential scanning calorimetry (DSC) was carried out with a Netsch STA 449 F1 Jupiter (Germany) under synthetic air with a heating rate of 10 k/min up to 1200 °C and combined with gas analysis during sample heating by quadrupole mass spectrometry (QMS 403 D, NETZSCH-Gerätebau GmbH, Selb, Germany).

The ionic conductivity of the powders was measured in the form of 5 mm diameter pellets (prepared as mentioned above) by an impedance analyzer, Solartron 1260, over a 1 Hz to 1 MHz frequency range. Before, thin conductive gold films were deposited on both sides of the sintered pellets by sputtering. To clarify, all impedance measurements were done at room temperature (~22 °C).

## 3. Results

First, we investigated the influence of the solvent mixture on nanoparticle production using the example of LYZP doped with 20% yttrium. The most suitable solvent mixture was then selected before discussing the synthesis and characteristics of materials with lower and no yttrium doping.

### Characterization of (LY_0.2_ZP)_PA50_ and (LY_0.2_ZP)_EA50_ Nanoparticles

**Figure 1:**
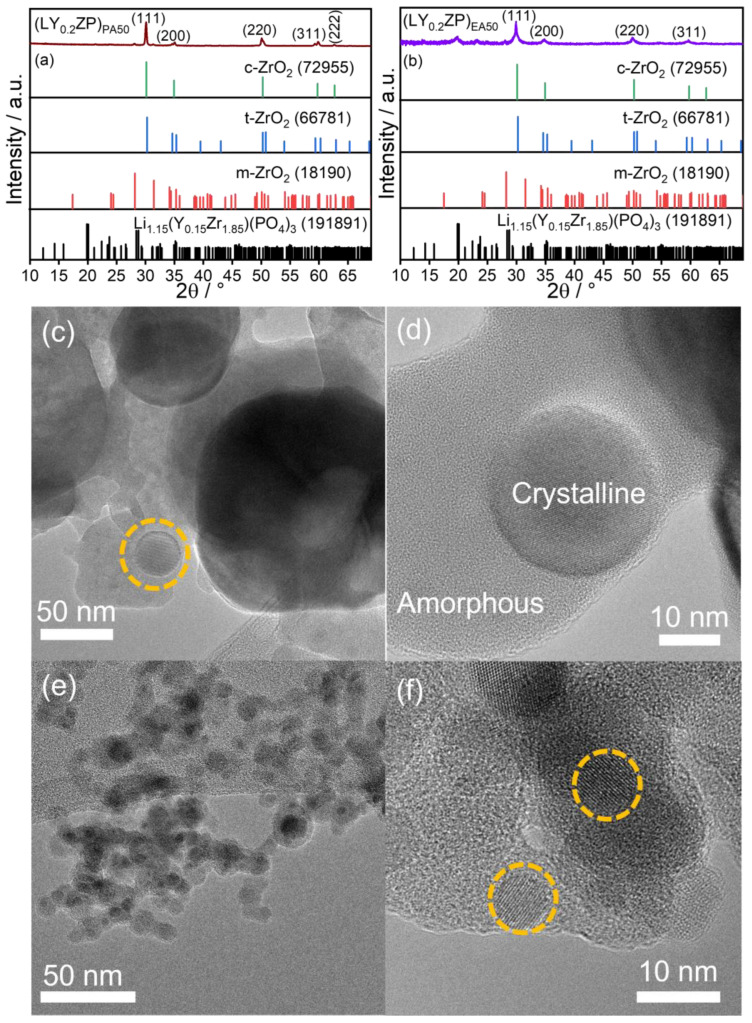
Phase composition and particle size of as-synthesized (LY_0.2_ZP)_PA50_ nanoparticles were determined by XRD (**a**), TEM (**c**), and HRTEM (**d**), respectively. Similarly, for (LA_0.2_ZP)_EA50_, the same was determined by XRD (**b**), TEM (**e**), and HRTEM (**f**), respectively. The yellowish circles highlight the single crystalline structures of as-synthesized samples.

To determine the phase compositions of as-synthesized (LY_0.2_ZP)_PA50_ and (LY_0.2_ZP)_EA50_, nanoparticles were analyzed with X-ray diffraction. The XRD patterns presented in Figure 1a,b indicate that both as-synthesized powders consist of more than one phase. It was not possible to conclusively determine whether the primary phase (main signal at 30.1°) of as-prepared (LY_0.2_ZP)_PA_ and (LY_0.2_ZP)_EA_ samples is tetragonal ZrO_2_ (t-ZrO_2_, ICSD 66781, space group P 42/n m c Z) or cubic ZrO_2_ (c-ZrO_2_, ICSD 72955, space group F m -3 m) due to the peak broadening and overlapping [75]. In the case of (LY_0.2_ZP)_PA50_ (Figure 1a), a splitting of peaks at 35° (220) and 60° (311) (indicating t-ZrO_2_) was observed; however, the splitting of peaks at 50° (indicating both c- and t-ZrO_2_) is not clear enough to be evaluated [76]. Furthermore, traces of the monoclinic zirconia phase (ICSD 18190, space group P1 21/c 1) at 28° and 31° were observed as an additional phase. In addition, the presence of monoclinic Li_2_CO_3_ (ICSD 66941, space group C1 2/c 1) at 21.3° can be cross-validated with the results from other measurement methods such as TGA and XPS, as will be shown later. It must be concluded that the intended phase of α-LYZP was not observed in the pristine particles made from propanol/propionic acid solution. In contrast, additional peaks at 20° and 23° were observed for particles made from ethanol/2-ethylhexanoic acid (LY_0.2_ZP)_EA50_, as shown in Figure 1b. It indicates the appearance of a new phase compared to (LY_0.2_ZP)_PA50_, which can most probably be assigned to the composition of a Y-doped LiZr_2_(PO_4_)_3_ (ICSD 191891). The peak broadening compared to (Figure 1a) is because the product from (LY_0.2_ZP)_EA50_ has a significantly lower crystallinity, which applies in particular to the signals for the new phase.

Prior to further analysis, the particle morphology and size distribution of as-synthesized (LY_0.2_ZP)_PA50_ and (LY_0.2_ZP)_EA50_ particles were carried out using TEM (Figure 1c,e) and HRTEM (Figure 1d,f), respectively. Both nano- and sub-micron particles were observed for particles produced from PrOH/PA (Figure 1c). The yellowish circles (Figure 1c) highlight the single crystalline structures of as-synthesized samples. We observed that, in contrast to EtOH/2-EHA, the use of PrOH/PA as solvent (LY_0.2_ZP)_PA50_ results in products with a significantly broader range (Figure S1a) [75]. In terms of mass, the large particles are important, but due to an insufficient number, it was not possible to fit the particle size distribution of these large particles in a meaningful way. The obtained size distribution for the smaller mode was plotted and fitted with a lognormal function (Figure S1a). The count median diameter (CMD) of this smaller mode is 16.6 nm with a geometric standard deviation ($\sigma_{g}$) 1.9. Due to the large mass fraction of big particles, it is obvious that the fitted CMD for the small mode does not agree with the estimated BET-based mean particle size, assuming a monodisperse spherical particle size. Thus, the measured specific surface area (SSA) of 5.74 m^2^/g corresponds to an average size of around 180 nm, mainly reflecting the big particle mode.

Srinivasan et al. have concluded that the particle sizes of t-ZrO_2_ and m-ZrO_2_ range from 2 to 20 nm and 20 to 70 nm, respectively [77]. Based on that, the size distribution of as-synthesized particles from (LY_0.2_ZP)_PA_ is assumed as a combined multinary model. To show the particle size distribution of as-synthesized particles from the experiment (LY_0.2_ZP)_PA50_ in high resolution, identical counted data from Figure 1c,d in the range from 2 nm to 50 nm are shown in Figure S1a,b. Figure 1e shows TEM images of as-synthesized materials from EtOH/2-EHA (LY_0.2_ZP)_EA50_ showing a significantly more homogeneous, monomodal particle size distribution, and no particles larger than 50 nm were observed (Figure 1e). The areas highlighted in Figure 1f show a single crystalline structure of zirconia and/or α-LYZP. Unlike the multimodal size distribution from (LY_0.2_ZP)_PA50_, the histogram of pristine (LY_0.2_ZP)_EA50_ shows a monomodal size distribution and could be fitted to a CMD of 7.8 nm with a geometric standard deviation $\sigma_{g}$ of 1.54 (Figure S1b). This result is in very good agreement with the BET-based mean particle size of 8.9 nm, see Table S2.

Besides the crystalline areas, the TEM images in Figure 1d suggest huge amounts of amorphous materials surrounding the crystalline particles. For further inspection, the compositions of the pristine materials were analyzed by XPS. On the one hand, it was possible to determine that the elements used are present in the product and, on the other, to clarify their chemical composition. For (LY_0.2_ZP)_PA50_, Figure S2a–c indicates the presence of Li_2_CO_3_. Detailed analysis of the P 2p spectra (Figure S2d) shows that phosphorus is present in the form of phosphate (134 eV binding energy, indicating tetrahedral PO_4_^3−^). The presence of Yttrium as oxide was proven on the basis of the binding energy contributions at 160.4 eV corresponding to Y 3d_3/2_ and 158.3 eV corresponding to Y 3d_5/2_ (Figure S2e). Similarly, the XPS spectrum of zirconium in Figure S2f reveals the presence of zirconium core level at 185.8 eV in the form of Zr 3d_3/2_, and a higher binding energy contribution at 183.4 eV in the form of Zr 3d_5/2_, respectively. It is difficult to distinguish between the tetragonal and monoclinic phases of ZrO_2_ via XPS. However, the higher binding energy position of the 3d_5/2_ spectra is well within the range of tetragonal ZrO_2_ (i.e., monoclinic ZrO_2_ generally appears at lower binding energy) [78]. Thus, this finding agrees with our XRD data. However, the shoulder interface spectra at ~181 eV are not observed for tetragonal ZrO_2_ in this case [78]. For (LY_0.2_ZP)_EA50,_ a significant peak at BE ~291 eV was detected in the spectrum of C 1s (Figure S3b), which indicates the presence of a carbonate bond. Since hints for crystalline Li_2_CO_3_ are almost missing in the XRD pattern, we assume that the pristine materials mainly contain amorphous Li_2_CO_3_ in addition to amorphous compounds of yttrium and phosphorous.

**Figure 2:**
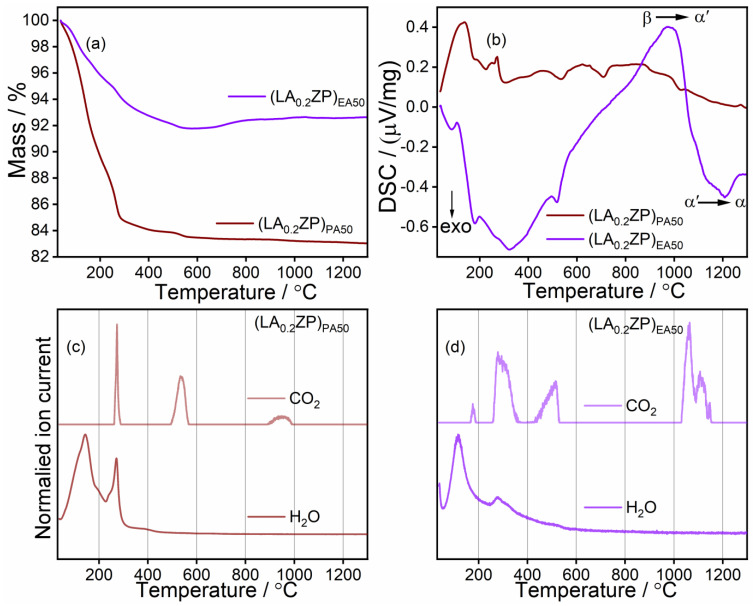
TGA and DSC of as-synthesized samples are shown in (**a**,**b**), respectively. (**c**,**d**) describe the CO_2_ and H_2_O QMS signal during TGA measurement of (LY_0.2_ZP)_PA50_ and (LY_0.2_ZP)_EA50_, respectively.

Further characterization of the as-synthesized (LY_0.2_ZP)_PA50_ and (LY_0.2_ZP)_EA50_ (Figure 2a,b) was performed with TGA/DSC/QMS analysis, with the bold line showing the TGA curve and the dotted line showing the DSC signal. The TGA curve demonstrates a total weight loss of around 17% and 8% for (LY_0.2_ZP)_PA50_ and (LY_0.2_ZP)_EA50_, respectively. The initial mass loss (~12% and ~3% for (LY_0.2_ZP)_PA50_ and (LY_0.2_ZP)_EA50_, respectively) between ambient temperature and <220 °C can be ascribed to the removal of physi- and chemisorbed H_2_O since only H_2_O was detected for both (LY_0.2_ZP)_PA50_ and (LY_0.2_ZP)_EA50_ via QMS (Figure 2c,d).

However, a slight amount of CO_2_ signal was detected for (LY_0.2_ZP)_EA50_ (Figure 2d) in the same region, indicating the thermal decomposition of physisorbed hydrocarbons, i.e., the carboxylic acids. The subsequent mass loss (<5% and ~1% for (LY_0.2_ZP)_PA50_ and (LY_0.2_ZP)_EA50_, respectively) between 230 °C and 400 °C can be attributed to the oxidation of higher hydrocarbons, which is accompanied by the release of CO_2_ and H_2_O. According to Egger et al., the next mass loss of around 500 °C for both the as-synthesized samples can be attributed to the decomposition of the residual Zr-precursor [79].

From the XPS and XRD studies, it is clear that the as-synthesized materials contain all the elements used in the precursor solution; however, most (Zr, P, Y) are present as an amorphous phase, and the material requires thermal annealing to reach the desired LYZP phase. From the DSC measurements, it can be deduced that the endothermic signal at ~980 °C for (LY_0.2_ZP)_EA50_ (Figure 2b) is most probably caused by the formation of a new phase (Equation (1), [43,44,74]), presumably triggered by the melting of lithium carbonate [80]. Furthermore, the exothermic process at about 1200 °C indicates a phase transformation (Equation (2), [43,44,74]) after the thermal decomposition of leftover lithium carbonate, releasing CO_2_. The slight increase in mass in the case of (LA_0.2_ZP)_EA50_ (<800 °C) may be due to a slight oxidation of the sample, which is supported by the DSC measurement showing an exothermic signal at about 550 °C.

(1) $$\beta \to \alpha '\left(\sim 1000\;{}^{\circ}C\right)$$

(2) $$\alpha ’\to \alpha \left(\sim 1200\;{}^{\circ}C\right)$$

**Figure 3:**
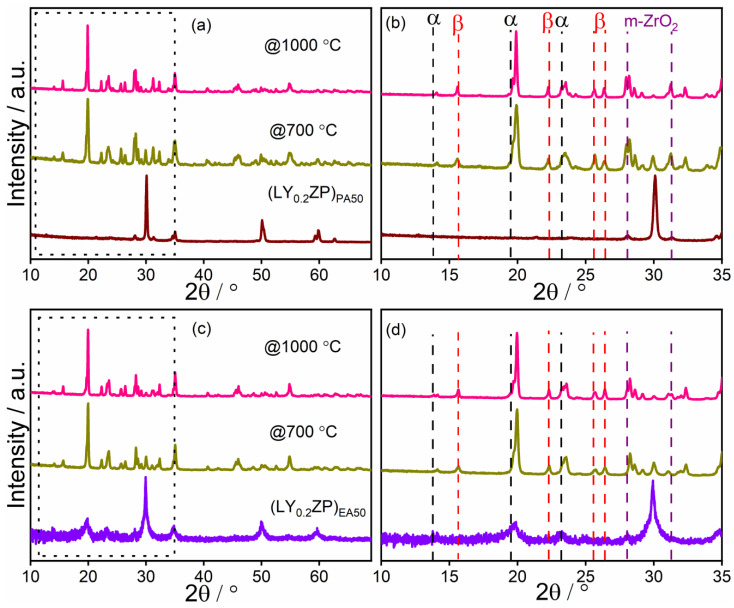
(**a**,**c**) XRD patterns comparison before and after annealing of (LY_0.2_ZP)_PA50_ and (LY_0.2_ZP)_EA50_, respectively, at different temperature conditions for 1 h under O_2_. (**b**,**d**) corresponding detailed illustration in the range of 10° to 35° 2θ, α refers to rhombohedral phase Li_1+x_Y_x_Zr_2−x_(PO_4_)_3_ and β refers to orthorhombic phase Li_1+x_Y_x_Zr_2−x_(PO_4_)_3_.

As discussed above, the phase change occurs for both the as-synthesized samples at relatively higher temperatures. Thus, the as-prepared materials were annealed at 700 and 1000 °C in a tube furnace under oxygen, with subsequent analysis of the crystal structure. Figure 3a,c reveals the effect of calcination on as-synthesized samples at different temperatures. When the pressed pellet from (LY_0.2_ZP)_PA50_ was heated at 700 °C for 1 h, XRD patterns showed a significant difference compared to the XRD pattern of pristine nanoparticles (Figure 3a). At this point, a significant decrease in the overall presence of t-ZrO_2_ can be observed. Moreover, a sharp peak at 20° appears after annealing at 700 °C in the case of (LY_0.2_ZP)_PA50_, indicating the formation of the LiZr_2_(PO_4_)_3_ phase (Figure 3a). Although the decrease in the t-ZrO_2_ phase is welcomed, the intended rhombohedral phase has not been achieved. This further compels to anneal the samples at even higher temperatures.

The detailed illustration of the dotted rectangular in Figure 3a between 10° and 30° 2θ is shown in Figure 3b and Figure 3d for (LY_0.2_ZP)_PA50_ and (LY_0.2_ZP)_EA50_, respectively. Based on that, it can be determined that β phase (orthorhombic) LYZP was obtained at 700 °C as the primary phase in both of the as-synthesized samples. As stated above, the t-ZrO_2_ phase decreases significantly at 1000 °C. However, an increment in m-ZrO_2_ has been observed (Figure 3b,d). Furthermore, t-ZrO_2_ transforms into m-ZrO_2_ after annealing at 1150 °C for 1 h (peak at 28°) (Figure S4a,b) [81]. When the annealing temperature further increased to 1150 °C, peaks belonging to the (rhombohedral) α-LYZP phase at 2θ 14°, 19.5°, and 23° start to appear. Ultimately, part of β phase transforms α phase under the heating condition of 1300 °C for 1 h. Nevertheless, α and β phases of LYZP still coexist even after annealing at high temperatures (1300 °C) (Figure S4a–d). Similarly, the smaller peaks between 2θ = 40–60° can be attributed to the (rhombohedral) α-LYZP phase (2θ: 42, 42.6, 45.3, 49, 54.8°) and the β phase of LYZP (2θ: 40.6, 46.1°). It can be concluded that, in addition to high temperatures over 1150 °C, the transition of transformation from the β phase of LYZP to the α phase needs a longer dwell time [74].

Raman spectroscopy (Figure S5) is used as a complementary to XRD to confirm the phase change during the calcination of the (LY_0.2_ZP)_PA_ as-synthesized sample. Here it is to be noted that as-synthesized samples from SFS are generally covered with unburned residual carbonaceous components (Figure 1d), which hinders the regular Raman measurements. Thus, almost no Raman signals could be observed for as-synthesized samples. However, after annealing, the absorption bands of m-ZrO_2_ are easily recognized in Figure S5. Annealing at 1150 °C for 1 h results in extensive transformation of t-ZrO_2_ to the m-ZrO_2_ structure. All absorption bands in the range of 180 cm^−1^ to 616 cm^−1^ highlighted with dotted rectangular in Figure S5 refer to the monoclinic phase zirconia [82], while the main bands of t-ZrO_2_ at 250 cm^−1^ and 640 cm^−1^ are absent [83,84]. Due to the phase transition, tetragonal zirconia as a minor impurity has not been detected by Raman after annealing. A band at 1028 cm^−1^ was assigned to P-O stretching vibrations [85], which accounted for the presence of phosphate, confirming LYZP phase formation.

To determine the phase composition of (LY_0.2_ZP)_PA50_ after annealing at 1300 °C for 1 h under O_2_, the result of fitted data using Rietveld refinement is shown in Figure S4e. The XRD pattern from (LY_0.2_ZP)_PA50_ after annealing could be fitted well with the α-phase LYZP (ICSD 191891, space group R-3 c H). There is a substantial amount of β-Li_1.2_Y_0.2_Zr_1.8_(PO_4_)_3_ (ICSD 91113, space group P b n a) in the pellet after heat treatment. m-ZrO_2_ and t-ZrO_2_ were also observed as impurities. A peak at 21° might be ascribed to the presence of Y(PO_3_)_3_ (ICSD 420121, space group C 1 c 1). Based on the result after refinement, the contents of the rhombohedral α-LYZP and β-LYZP are 31.5% and 40.3%, respectively (Table 3).

As seen above, the phase transformation progress indicates the co-existence of α and β phase LYZP after annealing at 1300 °C for 1 h, even for (LY_0.2_ZP)_EA50_. Nevertheless, the sharpest peak in Figure S4d and Figure 3d after annealing at 1300 °C is at 19.8°, belonging to α-LYZP, while the sharpest peak for (LY_0.2_ZP)_PA50_ was at 28°, belonging to m-ZrO_2_ (Figure S4b and Figure 3b). However, m-ZrO_2_ (shown in purple dotted line, Figure S4d) is still present as an impurity in the case of (LY_0.2_ZP)_EA50_ even after annealing at 1300 °C similar to (LY_0.2_ZP)_PA50_. The fitted XRD pattern in Figure S4f indicates that the primary phase of sintered material from (LY_0.2_ZP)_EA50_ is α-LYZP while the secondary phase is β-LYZP. Additionally, the composition of m-ZrO_2_ as a main impurity phase decreases significantly from 27.6 wt.% (in the case of (LY_0.2_ZP)_PA50_, Table 3) to 14.2 wt.% (Table 3; in the case of (LY_0.2_ZP)_PA50_). Correspondingly, the composition of α-LYZP increases from 31.5 wt.% to 49.6 wt.%. In contrast to (LY_0.2_ZP)_PA50_ Figure S4e, the results shown in Figure S4f testify to the potential of solvent ‘B’ to form desirable homogeneous nanoparticles of α-LYZP with higher purity.

Based on further study of literature [72], and at this point to further increase α- phase, the volumetric concentration of 2-ethylhexanoic acid (2-EHA) (in solvent mixture ‘B’) was increased to 70 vol.% in the experiment denoted as (LY_0.2_ZP)_EA70_ to investigate its corresponding effect on the composition development after heat treatment.

**Figure 4:**
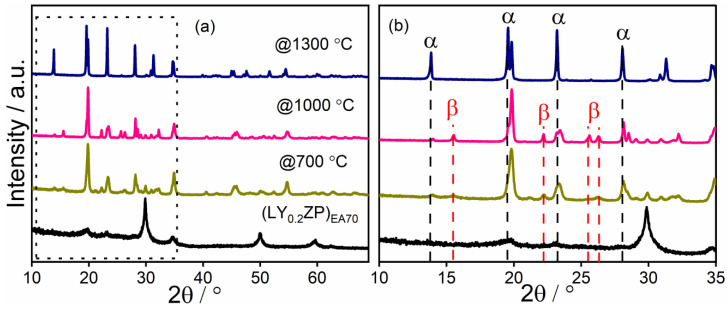
(**a**) XRD patterns of materials from (LY_0.2_ZP)_EA70_ before and after annealing at different temperature conditions for 1 h under O_2_. (**b**) corresponding detailed illustration in the range of 10° to 35° 2θ. α and β refer to the rhombohedral phase and orthorhombic.

**Figure 5:**
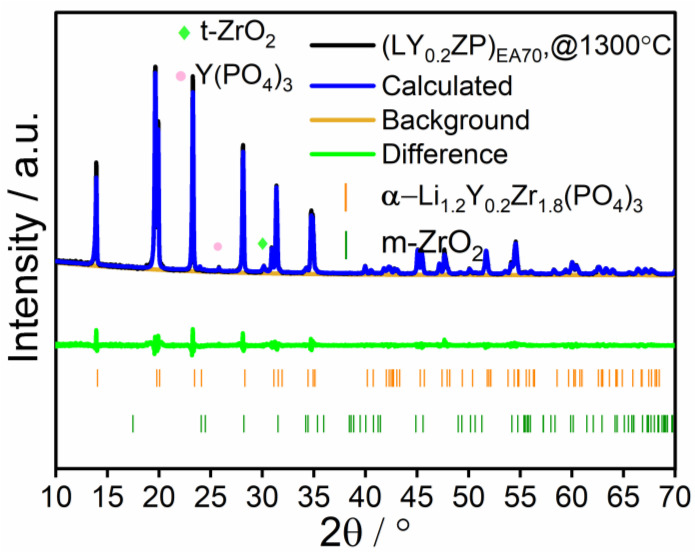
Fitted data of XRD pattern from (LY_0.2_ZP)_EA70_ after annealing at 1300 °C for 1 h under O_2_.

The as-synthesized samples were named (LY_0.2_ZP)_70_, keeping the Y^3+^ doping as constant as the previous (LY_0.2_ZP)_EA50_. The XRD pattern of (LY_0.2_ZP)_EA70_ is shown in Figure 4, and it shows almost no visible difference from (LY_0.2_ZP)_EA50_ sample. A significant difference illustrated in Figure 4b is that β-LYZP was not detected in the XRD pattern after annealing at 1300 °C for 1 h under O_2_. An increased concentration of 2-EHA may be beneficial to obtain α-LYZP with higher purity. According to Rietveld refinements of the XRD patterns shown in Figure 5, α-LYZP is present as the primary phase, while m-ZrO_2_ is the secondary phase. Impurities such as t-ZrO_2_ and Y(PO_4_)_3_ (ICSD 79754, space group I 41/a m d Z) were observed. The stoichiometric setup of the experiment might have an influence on the presence of impurity ZrO_2_ and Y(PO_4_)_3_ after heat treatment at 1300 °C. The loss of lithium during high-temperature SFS and subsequent annealing might result in the presence of impurities such as Y(PO_4_)_3_ [63].

To summarize and show the effective manipulation of solvent mixture keeping Y^3+^ doping constant, a comparative XRD of (LY_0.2_ZP)_PA50_, (LY_0.2_ZP)_EA50_, (LY_0.2_ZP)_EA70_ graph is shown in Figure S7a. To obtain the α-Li_1.2_Y_0.2_Zr_1.8_(PO_4_)_3_, heat treatment of pristine material above 1200 °C is inevitable in practice [44]. To illustrate the peak-intensive zone (highlighted with dotted rectangular in Figure S7a in the range of 10° to 35° 2theta more clearly), Figure S7b is shown. Compared to XRD patterns of materials after annealing from (LY_0.2_ZP)_PA50_ and (LY_0.2_ZP)_EA50_, a significant difference in XRD pattern after heat treatment from (LY_0.2_ZP)_EA70_ is the absence of β-LYZP. Regarding the improvement of the content of α-LYZP, the utilization of the solvent mixture ethanol/2-EHA (1:1 by volume) has a slight effect on composition after heat treatment at 1300 °C. On the other hand, an increased proportion of 2-EHA from 50 vol% to 70 vol% has a significant effect on composition after heat treatment at 1300 °C. To better understand this, the composition of materials after annealing at 1300 °C for 1 h under O_2_ from cases (LY_0.2_ZP)_PA50_, (LY_0.2_ZP)_EA50,_ and (LY_0.2_ZP)_PA50_ was shown in Table 4 via Rietveld refinement.

The most attractive observation is that the crystalline content of α-LYZP of material from an experiment (LY_0.2_ZP)_EA70_ after sintering is 94.7 wt.%, which indicates the beneficial potential of employed solvent mixture ethanol/2-EHA (3:7 by volume). Again, one issue that must be mentioned is that the calculation of crystalline content based on Rietveld refinement is used qualitatively.

Compared to solvent mixture ‘A’, solvent mixture ‘B’ has a higher boiling point (Table S1), which might lead to a gas-to-particle mechanism. Moreover, the esterification reaction was found in a solution containing metal nitrates, ethanol, and 2-EHA [72]. Moreover, this in turn results in a gas-to-particle formation pathway leading to smaller nanoparticles [86]. Furthermore, it has been shown that the breakup mechanism of microexplosion during spray remains the same as that of a single droplet [87]. Thus, it indicates that precursor chemistry (e.g., the boiling point of the final solvent mixture, melting point of the solutes, chemical stability of the precursors, etc.) affects not only the behavior of the micro explosion but also the final synthesized materials. The decomposition of zirconium(iv) propoxide above 600 °C may influence the composition in the high-temperature synthesis of α-LYZP above 1000 °C.

## 4. Discussion

In our synthesis conditions, a significant amount of zirconium propoxide was decomposed into product zirconia which was not incorporated into the LYZP phase. So, to summarize, solvent ‘B’ encourages the micro-explosion/droplet breakup/fragmentation, leading to a very small size of the final droplets. Due to the low melting point of Li, Y percussor leads to fast and foremost vaporization. On the other hand, having a high melting point of Zr, the formation of zirconia will be the first step on the route of condensation & nucleation from high spray-flame temperature. The smaller the final droplet, resulting in smaller the zirconia in size. At last, other species will nucleate surrounding zirconia Figure 1d forming an amorphous layer. The smaller the size higher the sintering activity leading to better phase composition. The same logic applies to why EHA/EtOH (7:3/*v*:*v*) produces a much better phase after annealing than solvent ‘B’.

**Figure 6:**
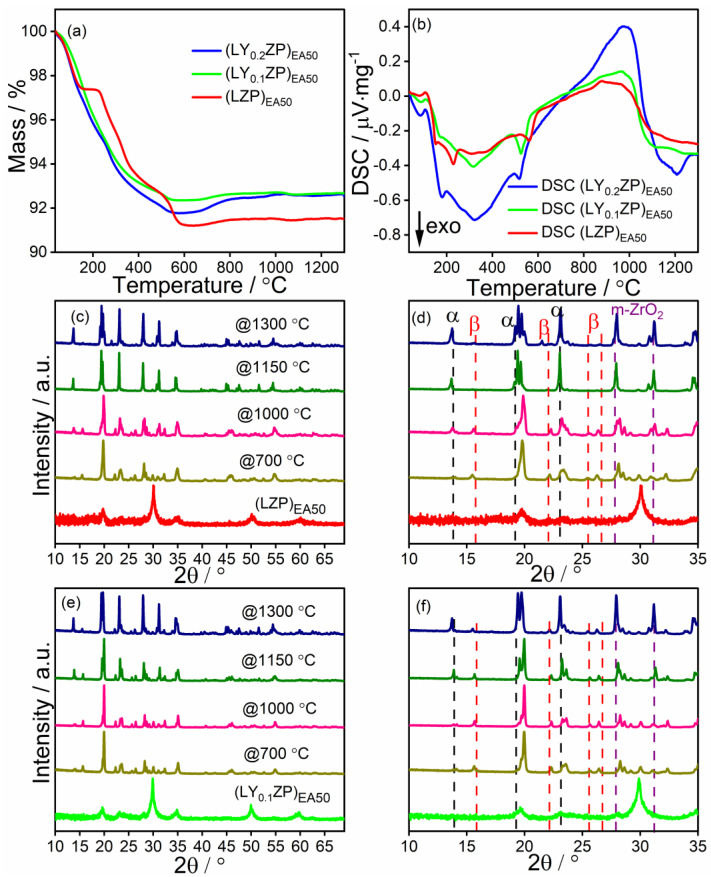
(**a**) TGA analysis of particles from (LY_0.2_ZP)_EA50_, (LY_0.1_ZP)_EA50_, and (LZP)_EA50_. (**b**) corresponding DSC analysis. (**c**) XRD patterns of materials from (LZP)_EA50_ before and after annealing at different conditions. (**d**) corresponding detailed illustration in the range of 10° to 35° 2θ. (**e**) XRD patterns of materials from (LY_0.1_ZP)_EA50_ before and after annealing at different temperature conditions for 1 h under O_2_. (**f**) corresponding detailed illustration in the range of 10° to 30° 2θ. α and β refer to the rhombohedral and orthorhombic phases of Li_1+x_Y_x_Zr_2−x_(PO_4_)_3_, respectively.

To investigate the effect of doped Y^3+^ concentration on Li _1+x_Y_x_Zr_2−x_(PO_4_)_3_ morphology involving solvent mixture ‘B’, experiments are denoted (LY_0.2_ZP)_EA50_, (LY_0.1_ZP)_EA50_, and (LZP)_EA50_, respectively. In this section, the effect of doping (varying Y concentration) has been discussed. As shown in Figure 6a,b, the thermal properties of the LY_x_ZP are comparable to each other. The first mass loss was due to physisorbed unburned precursors and later may be due to Li_2_CO_3_ degradation [63]. Moreover, the exothermic change ~1200 °C might be due to phase change which is a similar property to (LY_0.2_ZP)_PA50_ (Figure 2).

The phase composition of as-prepared LYxZP particles involving the solvent mixture ethanol/2-EHA (1:1 by volume) was determined with XRD in an overview way (Figure S6a). Similarly, the primary phase of as-synthesized materials from three experiments is t-ZrO_2_, and the secondary phase is m-ZrO_2_. Nevertheless, in contrast to the as-synthesized material from (LY_0.2_ZP)_PA50_, the significant presence of peaks at 20 °C and 23 °C (marked with a point-point-dash line) was observed in Figure S7a. It indicates that the utilized solvent mixture of ethanol and 2-EHA results in the presence of a new phase, which may be the expected Y-doped LiZr_2_(PO_4_)_3_ (ICSD 191891, Figure S7a).

At this point, it has been established that solvent mixture ‘B’ results in smaller nanoparticles with a narrower particle size distribution as well as a better phase composition than solvent mixture ‘A’. However, it is unclear if Y incorporation has any subsequent effect on phase composition. XRD results of (LZP)_EA50_ and (LY_0.1_ZP)_EA50_ are shown in Figure S6a and Figure 6b,e, respectively. The XRD patterns of materials from (LY_0.1_ZP)_EA50_ in Figure 6e show a similarity of the phase transformation as the case LY_0.2_ZP described in Figure 3c and Figure 5c. No β′-LYZP was detected in XRD patterns after annealing at 1300 °C after 1 h under O_2_. However, peak splitting at 20° 2θ in the XRD profile from (LZP)_EA50_ (Figure 6c,d) at a higher annealing temperature (i.e., 1300 °C) was observed. A similar kind of peak splitting has not been observed in any other samples, even after annealing at 1300 °C. There might be another reaction when the annealing temperature reaches 1300 °C. Unfortunately, after an intensive investigation of the literature, reasonable assumptions and convincing explanations are not supportive of this phenomenon. Further investigation of the mentioned splitting peak needs to be done and is beyond this work. 

It is clear from Figure 6c–f that the peak intensity of the α phase increases with increasing Y^3+^ doping. However, m-ZrO_2_ impurity remains within the system with varying Y^3+^ doping. The high-resolution XPS results of P 2p, Zr 3d, and Y 3d elements are shown in Figure S6b–d, respectively. All three P 2p peaks from the experiment involving solvent mixture ‘B’ demonstrate a symmetric shape, which manifests the presence of one type of phosphorus (i.e., phosphate). The peaks related to the BE of ~183.2 eV and ~185.6 eV (Figure S6c), pointed with arrows, correspond to Zr 3d5/2 and Zr 3d3/2, while peaks related to the BE of ~158.1 eV and ~160.1 eV (Figure S6d, pointed with arrows) correspond to Y 3d5/2 and Y 3d3/2, respectively. XPS spectra of Y and Zr from experiments (LY_0.2_ZP)_EA50_, (LY_0.1_ZP)_EA50_, and (LZP)_EA50_ in Figure S6c, d demonstrate the structure change of the bonds P-O-Zr and P-O-Y as a different compositions of aliovalent yttrium ion were incorporated.

**Figure 7:**
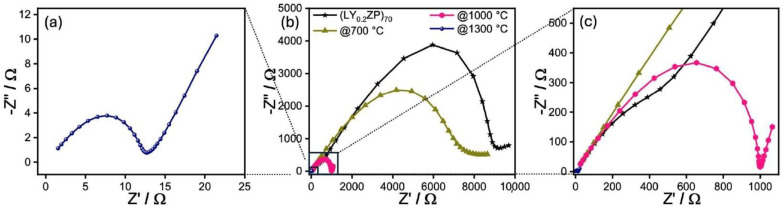
Impedance spectra of as-synthesized (LY_0.2_ZP)_EA70_ and after annealing at different temperatures. Figure (**b**) shows an overview of all spectra, (**a**,**c**) are enlarged sections of the measurements on samples after annealing at 1300 and 1000 °C, respectively.

After quantitative analysis via Rietveld refinement, there is no evidence that shows materials after annealing at 1300 °C from cases LY_0.2_ZP)_EA50_, (LY_0.1_ZP)_EA50_, and (LZP)_EA50_ consist of any triclinic phase LYZP (α′-LYZP). Fitted data via Rietveld refinement of materials after annealing at 1300 °C from cases LZP is shown in (Figure S8). The impedance spectra of (LY_0.2_ZP)_70_ and (LZP)_EA50_ particles after annealing at different temperatures are shown in (Figure 7) and (Figure S8), respectively. Both the as-synthesized samples show the highest resistance at room temperature compared to annealed samples.

The impedance of the as-synthesized (LY_0.2_ZP)_70_ is reduced by almost three orders of magnitude by annealing at 1300 °C, although the shape of the spectra remains almost the same (Figure 7a–c). As known from the literature [88], the impedance spectrum of NASICON-type structures exhibits relaxation processes for the bulk and grain boundary conductivity, which can be seen in two more or less pronounced semicircles in the higher-frequency part of the spectrum. In the low-frequency part, the spectrum is characterized by the capacitive properties of the ionic conductor, which manifest themselves in a straight line whose imaginary part of the impedance increases with decreasing frequency. This can be seen particularly well for the material tempered at 1000 °C (Figure 7c) and 1300 °C (Figure 7a). This low-frequency behavior is also characteristic of pure ionic conductors. We attribute this change in impedance to the fact that t-ZrO_2_ transforms into the NASICON structure with increasing annealing temperature. This, combined with significant grain growth, leads to a highly increased ionic conductivity compared to the as-synthesized samples. And true to our XRD analysis (Figure 4), Figure 7a indicates that after annealing at 1300 °C, (LY_0.2_ZP)_EA_ shows the best conductivity (1.14 × 10^−5^ S cm^−1^), which is comparable to the reported value [44]. In comparison, the undoped sample shows poor performance at similar conditions (Figure S8, 1.88 × 10^−6^ S cm^−1^).

## 5. Conclusions

Here, we have successfully synthesized nanocrystalline rhombohedral (α) lithium yttrium zirconium phosphate (LYZP) based on a spray-flame process followed by a short annealing step. In this case, the addition of Y^3+^ as a dopant is a mandatory step to stabilize the α phase. We have witnessed that direct synthesis of LYZP is not possible due to the shorter residual time and rapid nucleation of tetragonal zirconia (t-ZrO_2_) in contrast to species containing Li, Y, and phosphate species during gas-phase synthesis. This reaction mechanism is universal with different solvent mixtures (e.g., 2-ethyl hexanoic acid/ethanol, propanol/propanoic acid). It is shown that the 2-ethyl hexanoic acid/ethanol solvent mixture (‘B’) is suitable to synthesize nanoparticles with narrow size distribution with higher specific surface area compared to the nanoparticles synthesized by solvent mixture propanol/propanoic acid (‘A’). All products, in the case of A, annealed at <1300 °C exhibited the undesirable, poorly ion-conducting β phase with a mixture of a smaller percentage α phase. However, the particularly good mixing of t-ZrO_2_ and Li, Y, phosphate precursor, as occurs in spray flame synthesis, with appropriate Y^3+^ doping and solvent mixture ‘B’ shows advantages. To the best of our knowledge, this is the only bottom-up approach for α-LYZP synthesis.

## Figures and Tables

**Table 1:** Scheme of precursors and solvents and their corresponding nomenclature.

Nomenclature	Precursors				Solvents	
	Li	Y	Zr	P	(A)	(B)
	LiNO_3_ (50% excess Li)	Y(NO_3_)_3_ ·6H_2_O	ZP	TBP	Propanol/Propionic Acid (PrOH/PA) *V*/*V*	Ethanol/2-Ethylhexanoic Acid (EtOH/2-EHA) *V*/*V*
(LZP)_EA50_	✓	0	✓	✓	×	1:1
(LY_0.1_ZP)_EA50_	✓	0.1	✓	✓	×	1:1
(LY_0.2_ZP)_PA50_	✓	0.2	✓	✓	1:1	×
(LY_0.2_ZP)_EA50_	✓	0.2	✓	✓	×	1:1
(LY_0.2_ZP)_EA70_	✓	0.2	✓	✓	×	3:7

**Table 2:** Spray-flame reactor operating conditions.

Operating Parameters						
Dispersion CH_4_ [slm]	Dispersion O_2_ [slm]	Pilot Flame CH_4_ [slm]	Pilot Flame O_2_ [slm]	Quench Gas Air [slm]	Coaxial Sheath Air [slm]	Reactor Pressure [mbar]
1	9	2	16	240	140	800–820

**Table 3:** Composition of phases before and after annealing of (LY_0.2_ZP)_EA50_ and (LY_0.2_ZP)_PA50._

	Status		Content of Phase [%]		
		t-ZrO_2_	m-ZrO_2_	α-LYZP	β-LYZP
(LY_0.2_ZP)_PA50_	As-synthesized	34.0	39.9	/	/
	@1300 °C	0.6	27.6	31.5	40.3
(LY_0.2_ZP)_EA50_	As-synthesized	21.8	63.5	14.7	/
	@ 1300 °C	0.5	14.2	49.6	35.6

**Table 4:** Composition development of materials after annealing at 1300 °C for 1 h under O_2_.

Nomenclature	Solvent Mixture	Composition [wt%]		
	Propanol/Propionic Acid (1:1 by Volume)	α-LYZP	β-LYZP	m-ZrO_2_
(LY_0.2_ZP)_PA50_	Ethanol/2-EHA (1:1 by volume)	31.5	40.3	27.6
(LY_0.2_ZP)_EA50_	Ethanol/2-EHA (3:7 by volume)	49.6	35.6	14.2
(LY_0.2_ZP)_EA70_	Propanol/propionic acid (1:1 by volume)	94.7	/	3.1

## Data Availability

Data can be made available upon request.

## Supplementary Materials

The following supporting information can be downloaded at: https://www.mdpi.com/article/10.3390/nano14151278/s1, Table S1: Physical properties of solvents and precursors; Figure S1: Particle size distribution of as-synthesized particles from case (LY_0.2_ZP)_PA50_ (a) and (LY_0.2_ZP)_EA50_ (b) respectively; Figure S2: Results of XPS measurements of (a) Li 1s. (b) C 1s. (c) O 1s. (d) P 2p. (e) Y 3d. (f) Zr 3d of as-synthesized (LY_0.2_ZP)_PA50_; Figure S3: Results of XPS measurements of (a) Li 1s. (b) C 1s. (c) O 1s of as-synthesized (LY_0.2_ZP)_EA50_; Figure S4: (a,c) XRD patterns comparison before and after annealing at 1150 & 1300 °C of (LY_0.2_ZP)_PA_ and (LY_0.2_ZP)_EA_ respectively, for 1 h under O2. (b,d) corresponding detailed illustration in the range of 10° to 35° 2θ, α refers to rhombohedral phase Li_1+x_Y_x_Zr_2−x_(PO_4_)_3_ and β refers to orthorhombic phase Li_1+x_Y_x_Zr_2−x_(PO_4_)_3_. (e,f) phase composition of material from (LY_0.2_ZP)_PA_ and (LY_0.2_ZP)_EA_ respectively using Rietveld refinement after annealing at 1300 °C for 1 h under O_2_; Figure S5: Raman spectroscopy of materials from (LY_0.2_ZP)_PA50_ after sintering at different temperature conditions for 1 h under O_2_. All absorptions bands in dotted rectangular indicates the presence of m-ZrO_2_, while the dotted line is attributed to P-O stretching; Figure S6: (a) Compositions of as-synthesized materials from experiments involving solvent mixture ethanol/2-EHA (1:1 by volume). Point-point-dash lines refer to new present peaks compared to the compositions of as-synthesized particles from case (LY0_.2_ZP)_PA50_. XPS results from (b) P 2p. (c) Zr 3d. (d) Y 3d from experiments involving solvent mixture ‘B’ with varying Y^3+^ doping; Table S2. Comparison of as-synthesized average particle size; Figure S7: (a) XRD patterns of materials from (LY_0.2_ZP)_PA50_, (LY_0.2_ZP)_EA50_ and (LY_0.2_ZP)_EA70_ (from bottom to top) after annealing at different temperature conditions for 1h under O_2_. (b) corresponding detailed illustration in the range of 10° to 35° 2θ. α and β refers to rhombohedral phase and orthorhombic phase of Li_1+x_Y_x_Zr_2−x_(PO_4_)_3_ respectively; Figure S8: Impedance spectra of (LZP)EA50 particles after annealing at different temperatures (a). And (b) represents the zoomed image of the square in (a); Figure S9: (a) TEM (b) HRTEM of particles from (LY_0.1_ZP)_EA50_ (c) corresponding particle size distribution and fitted lognormal curve. (d) TEM (e) HRTEM of particles from (LZP)_EA50_ (f) corresponding particle size distribution and fitted lognormal curve.
