# Supplementary material for: Spray-Flame Synthesis of NASICON-Type Rhombohedral (α) Li1+xYxZr2−x(PO4)3 [x = 0–0.2] Solid Electrolytes
Source: Nanomaterials (Basel). 2024 Jul 30;14(15):1278. doi: 10.3390/nano14151278 (PMC11314149; doi:10.3390/nano14151278)
Supplement: Supplementary file 1 [file nanomaterials-14-01278-s001.zip › nanomaterials-3105180-supplementary.pdf]

nanomaterials

Article

Spray-Flame Synthesis of NASICON-Type Rhombohedral (α) $Li_{1+x}Y_xZr_{2-x}(PO_4)_3$ [x = 0–0.2] Solid Electrolytes

Md Yusuf Ali [1,†], Tianyu Chen [1,†], Hans Orthner [1] and Hartmut Wiggers [1,2,*]

[1] Institute for Energy and Materials Processes—Reactive Fluids, University of Duisburg-Essen, 47057 Duisburg, Germany; yusuf.ali@uni-due.de (M.Y.A.); tianyu-chen@marubeni.com (T.C.); hans.orthner@uni-due.de (H.O.)
[2] CENIDE, Center for Nanointegration Duisburg-Essen, 47057 Duisburg, Germany
* Correspondence: hartmut.wiggers@uni-due.de
† These authors contributed equally to this work.

Citation: Ali, M.Y.; Chen, T.; Orthner, H.; Wiggers, H. Spray-Flame Synthesis of NASICON-Type Rhombohedral (α) $Li_{1+x}Y_xZr_{2-x}(PO_4)_3$ [x = 0–0.2] Solid Electrolytes. *Nanomaterials* **2024**, *14*, x. https://doi.org/10.3390/xxxxx

Academic Editor: Jun-ho Yum

Received: 28 June 2024
Revised: 18 July 2024
Accepted: 24 July 2024
Published: 30 July 2024

Copyright: © 2024 by the authors. Licensee MDPI, Basel, Switzerland. This article is an open access article distributed under the terms and conditions of the Creative Commons Attribution (CC BY) license (https://creativecommons.org/licenses/by/4.0/).

Table S1. Physical properties of solvents and precursors

Chemical Formula	Category	Melting point [°C]	Boiling point [°C]
$LiNO_3$	Solute	255	600
$Y(NO_3)_3{\cdot}6H_2O$	Solute	52	Decomposes at 640
$Zr(NO_3)_4$ (ZN)	Solute	/	Decomposes at 100
$Zr^{x+}{\cdot}xH_3CCOOH$ (ZA)	Solute	/	/
$Zr(OCH_2CH_2CH_3)_4$ (ZP)	Solute	661	/
2-Propanol	Solvent	/	82
Propionic acid	Solvent	/	141
Ethanol	Solvent	/	78
2-Ethylhexanoic acid	Solvent	/	217
Acetic acid	Solvent	/	118

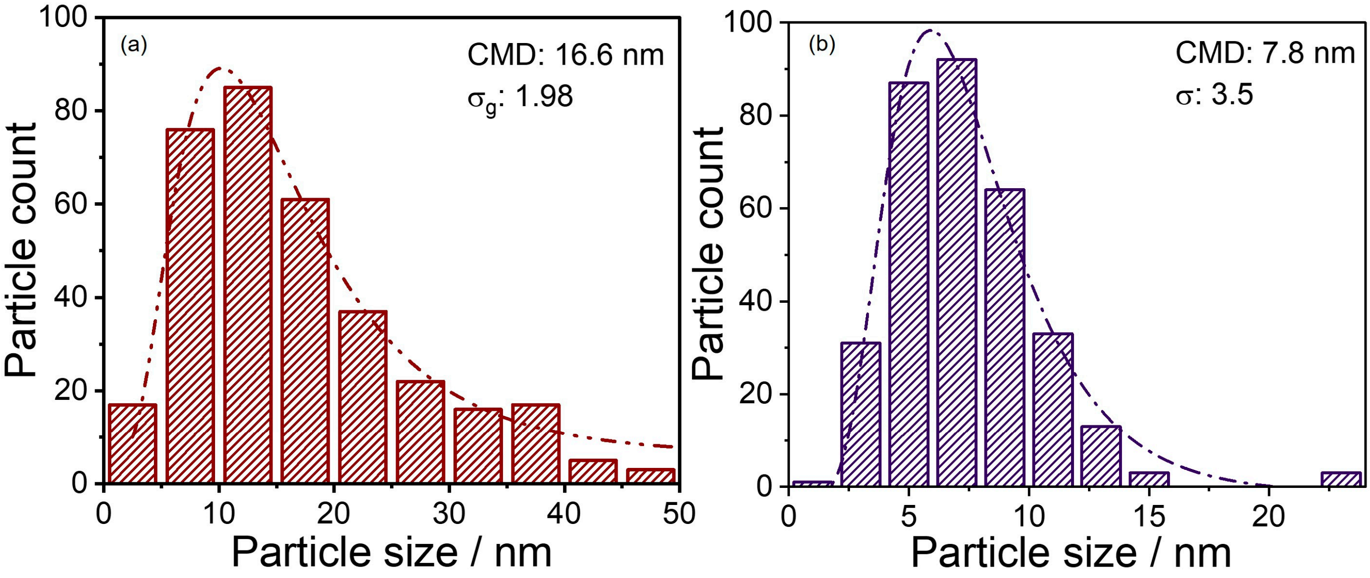

Figure S1. Particle size distribution of as-synthesized particles from case $(LY_{0.2}ZP)_{PA50}$ (a) and $(LY_{0.2}ZP)_{EA50}$ (b) respectively.

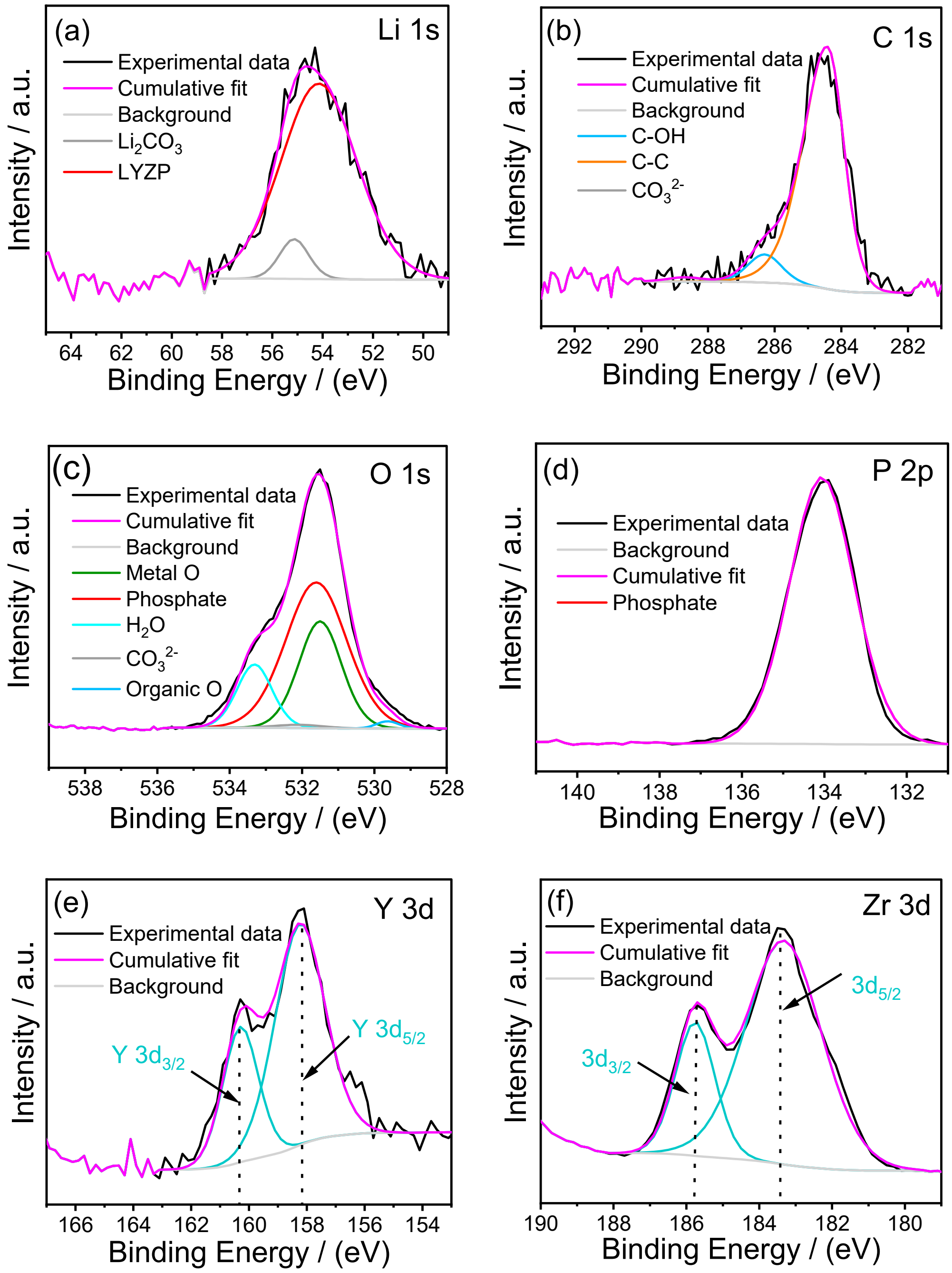

Figure S2. Results of XPS measurements of (a) Li 1s. (b) C 1s. (c) O 1s. (d) P 2p. (e) Y 3d. (f) Zr 3d of as-synthesized $(LY_{0.2}ZP)_{PA50}$

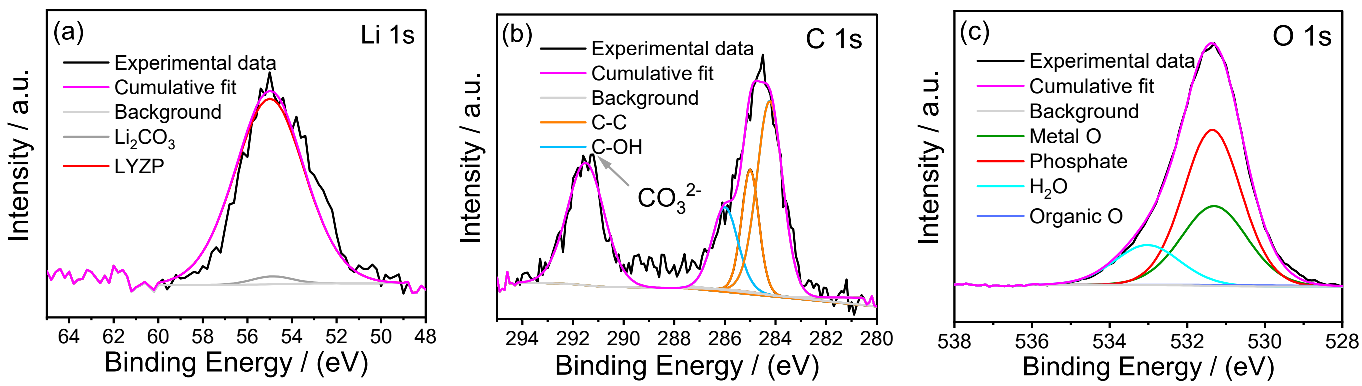

Figure S3. Results of XPS measurements of (a) Li 1s. (b) C 1s. (c) O 1s of as-synthesized $(LY_{0.2}ZP)_{EA50}$.

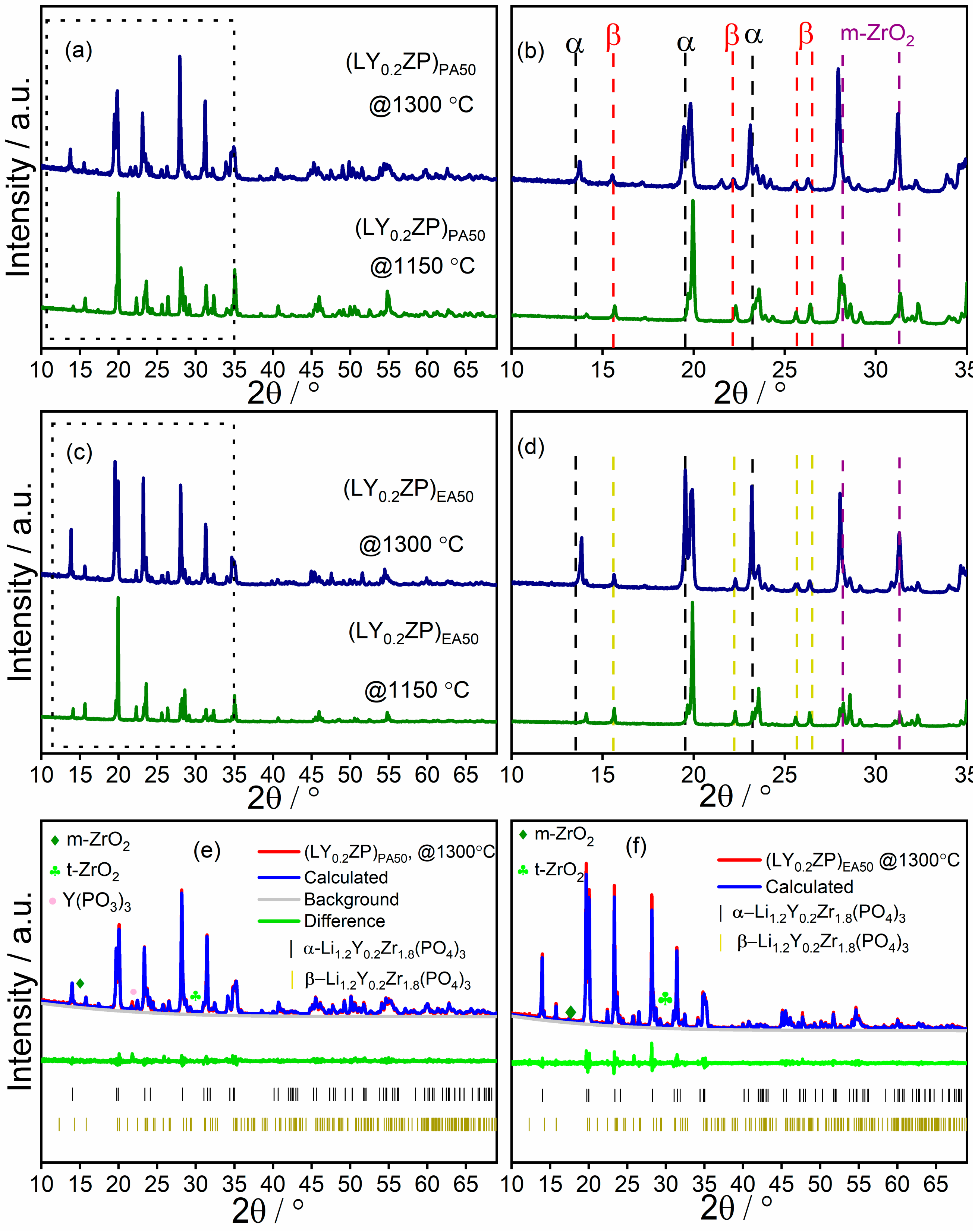

Figure S4. (a), (c) XRD patterns comparison before and after annealing at 1150 & 1300 °C of $(LY_{0.2}ZP)_{PA}$ and $(LY_{0.2}ZP)_{EA}$ respectively, for 1 h under O2. (b), (d) corresponding detailed illustration in the range of 10º to 35º 2θ, α refers to rhombohedral phase $Li_{1+x}Y_xZr_{2-x}(PO_4)_3$ and β refers to orthorhombic phase $Li_{1+x}Y_xZr_{2-x}(PO_4)_3$. (e), (f) phase composition of material from $(LY_{0.2}ZP)_{PA}$ and $(LY_{0.2}ZP)_{EA}$ respectively using Rietveld refinement after annealing at 1300 ºC for 1 h under O_2.

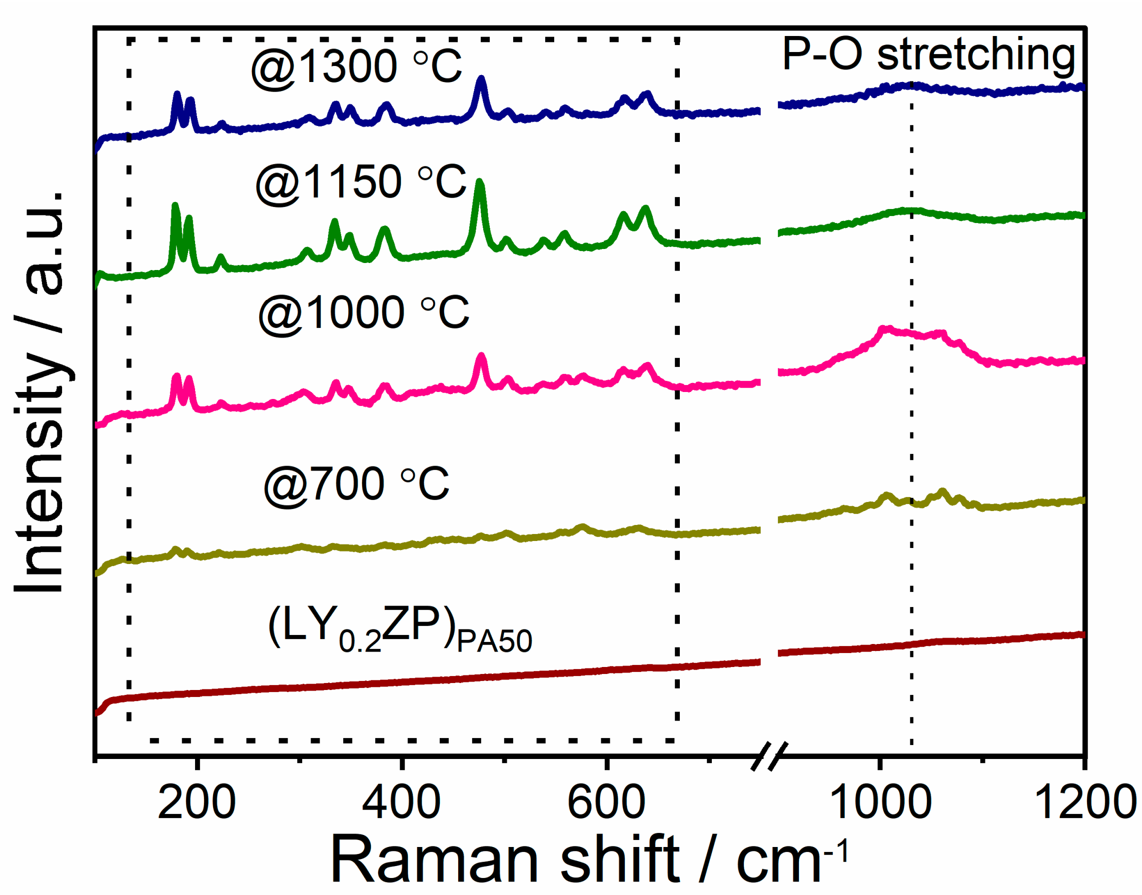

Figure S5. Raman spectroscopy of materials from $(LY_{0.2}ZP)_{PA50}$ after sintering at different temperature conditions for 1 h under O_2. All absorptions bands in dotted rectangular indicates the presence of m- ZrO_2, while the dotted line is attributed to P-O stretching.

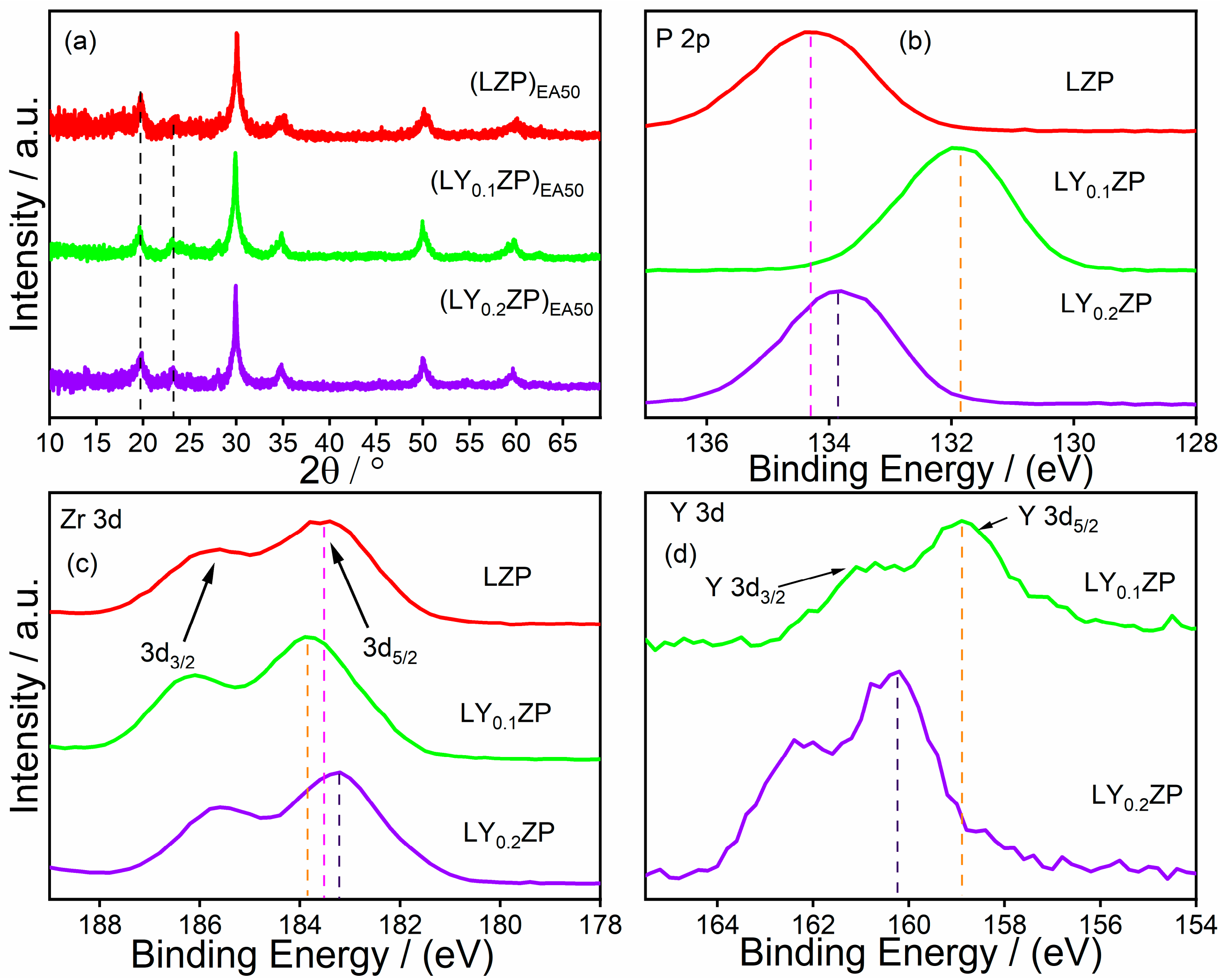

Figure S6. (a) Compositions of as-synthesized materials from experiments involving solvent mixture ethanol/2-EHA (1:1 by volume). Point-point-dash lines refer to new present peaks compared to the compositions of as-synthesized particles from case $(LY_{0.2}ZP)_{PA50}$. XPS results from (b) P 2p. (c) Zr 3d. (d) Y 3d from experiments involving solvent mixture 'B' with varying Y^{3+} doping.

Table S2. Comparison of as-synthesized average particle size

Nomenclature	As-synthesized average particle size [nm]			
	Specific Surface area [m²/g]	BET	TEM	Refinement (m-ZrO2)
$(LY_{0.2}ZP)_{PA50}$	5.7	180.6	25.7	27.2 ± 0.8
$(LY_{0.2}ZP)_{EA50}$	122.1	8.9	7.8	3.6 ± 0.7
$(LY_{0.1}ZP)_{EA50}$	129.0	8.4	7.8	14.8 ± 0.4
$(LZP)_{EA50}$	116.7	9.3	9.1	9.6 ± 5.5

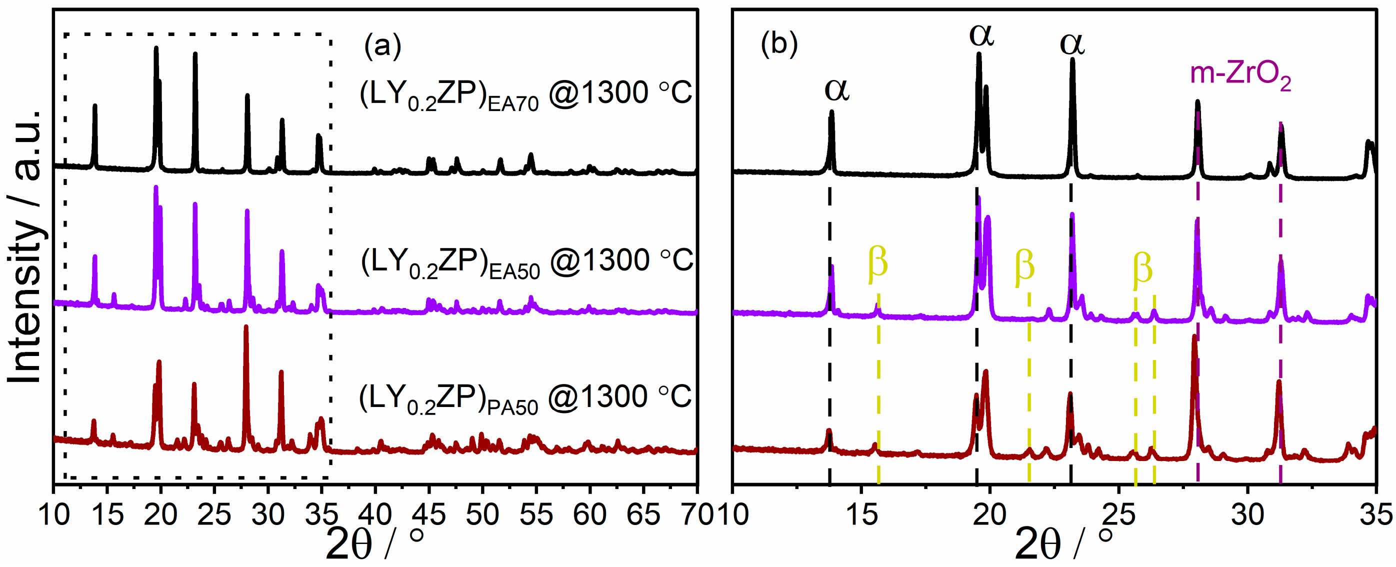

Figure S7. (a) XRD patterns of materials from $(LY_{0.2}ZP)_{PA50}$, $(LY_{0.2}ZP)_{EA50}$ and $(LY_{0.2}ZP)_{EA70}$ (from bottom to top) after annealing at different temperature conditions for 1h under O_2. (b) corresponding detailed illustration in the range of 10° to 35° 2θ. α and β refers to rhombohedral phase and orthorhombic phase of $Li_{1+x}Y_xZr_{2-x}(PO_4)_3$ respectively.

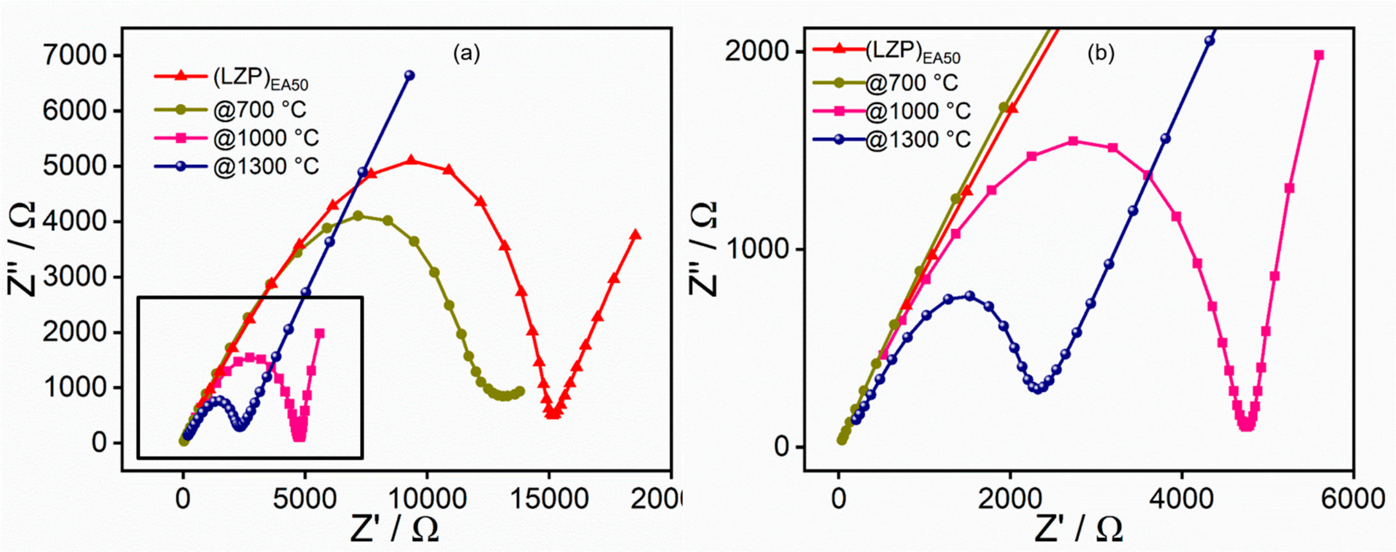

Figure S8. Impedance spectra of (LZP)EA50 particles after annealing at different temperatures (a). And (b) represents the zoomed image of the square in (a).

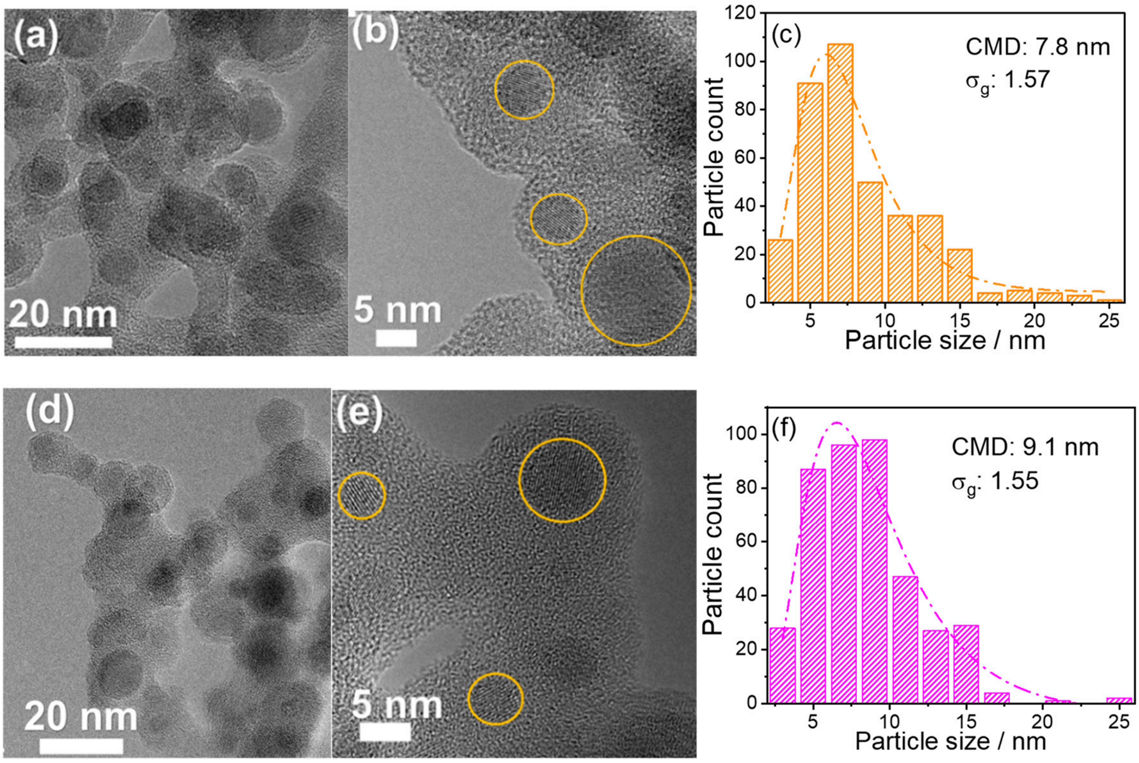

Figure S9. (a) TEM (b) HRTEM of particles from $(LY_{0.1}ZP)_{EA50}$ (c) corresponding particle size distribution and fitted lognormal curve. (d) TEM (e) HRTEM of particles from $(LZP)_{EA50}$ (f) corresponding particle size distribution and fitted lognormal curve.
